# Supplementary figures and images for: Analysis of the Rdr1 gene family in different Rosaceae genomes reveals an origin of an R-gene cluster after the split of Rubeae within the Rosoideae subfamily
Source: PLoS One. 2020 Jan 23;15(1):e0227428. doi: 10.1371/journal.pone.0227428 (PMC6977733; doi:10.1371/journal.pone.0227428)

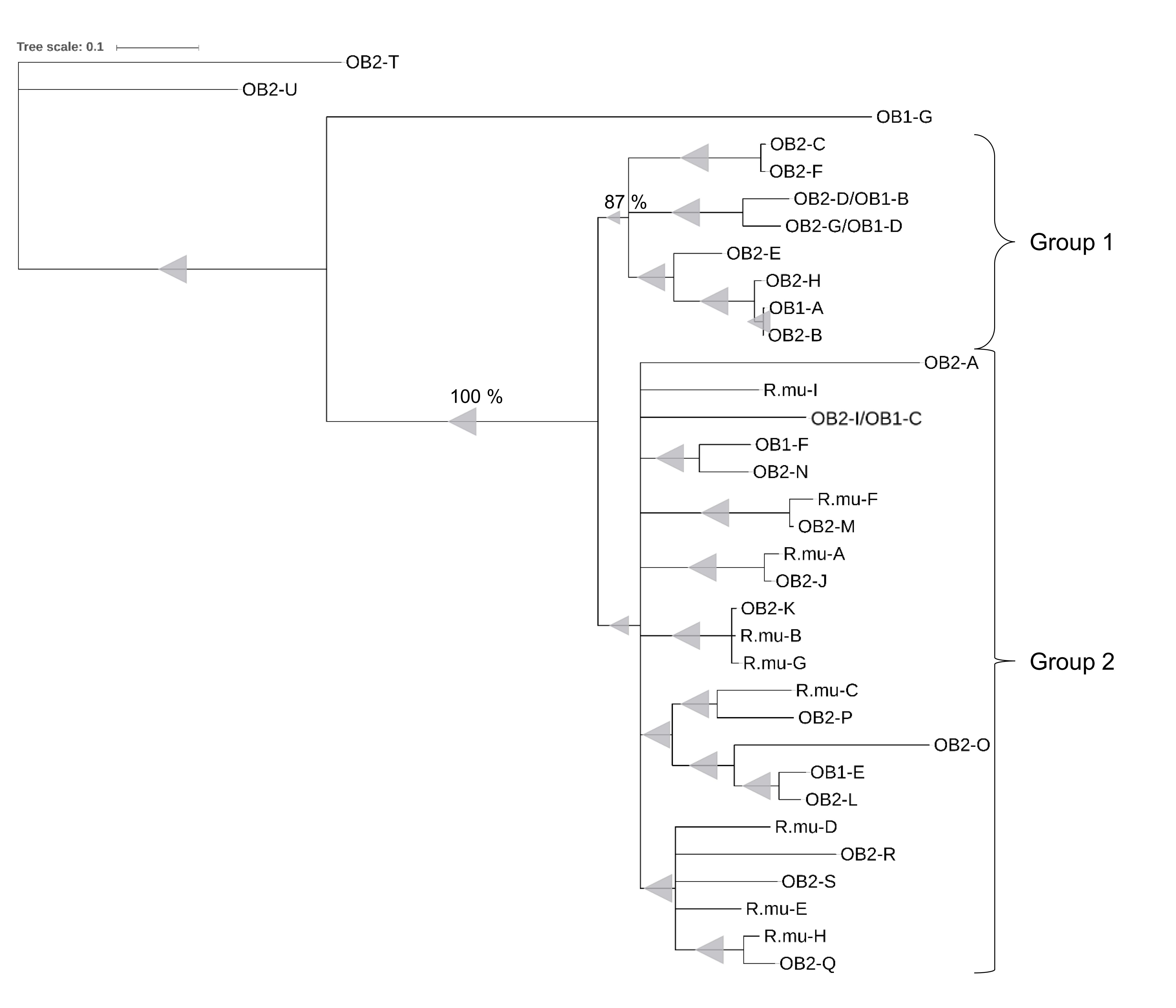

Supplement: S1 Fig — The maximum likelihood method based on the JTT matrix-based model was used to calculate the phylogenetic tree. A test of phylogeny was performed using the bootstrap method with 500 replicates. Branches reproduced in less than 75% of bootstrap replicates are collapsed. Bootstrap values are indicated as triangles, whereas the smallest value represents 87% and the largest 100%. (TIF) [file pone.0227428.s001.tif]

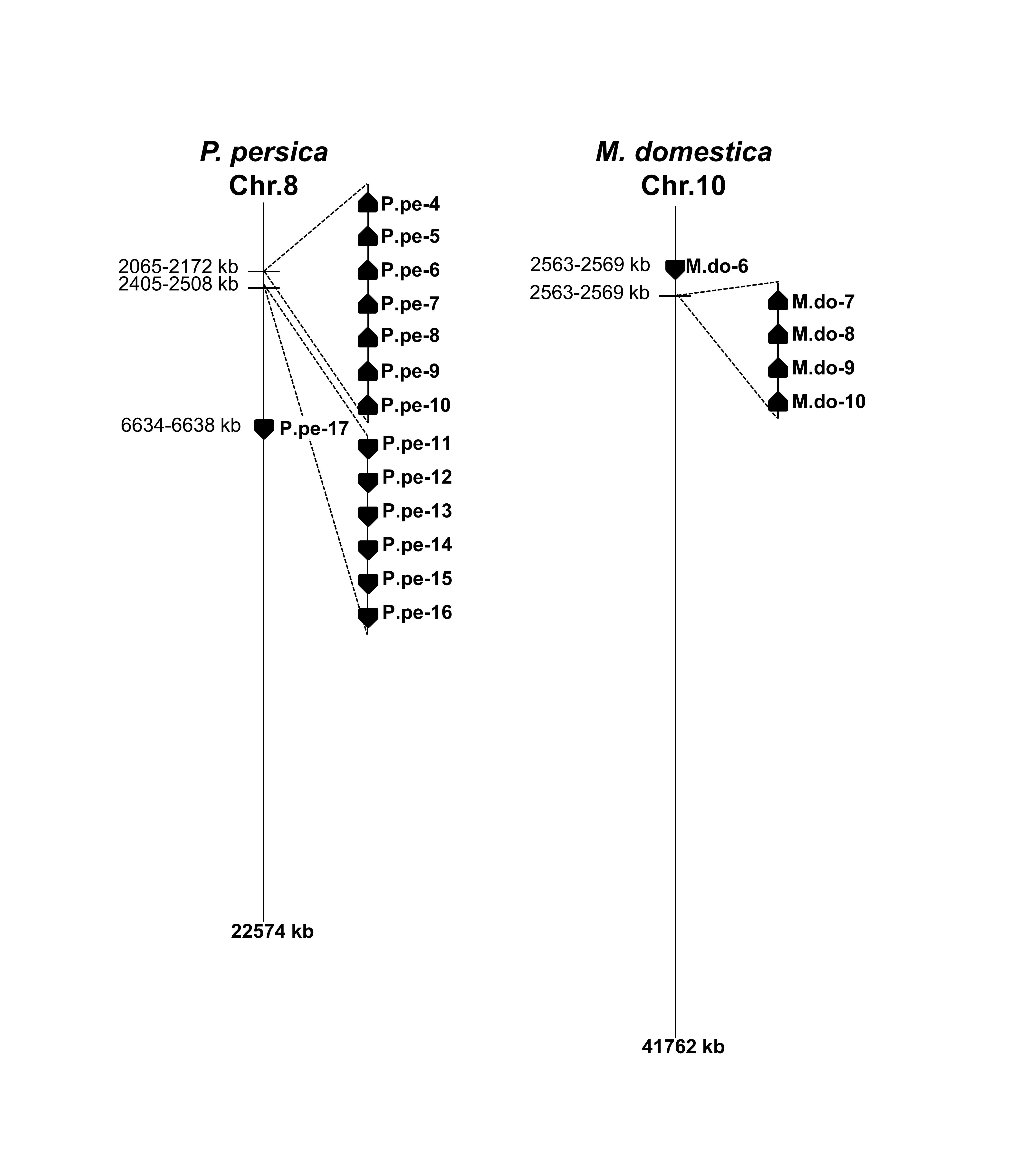

Supplement: S3 Fig — (TIF) [file pone.0227428.s003.tif]
