# Supplementary material for: Analysis of the Rdr1 gene family in different Rosaceae genomes reveals an origin of an R-gene cluster after the split of Rubeae within the Rosoideae subfamily
Source: PLoS One. 2020 Jan 23;15(1):e0227428. doi: 10.1371/journal.pone.0227428 (PMC6977733; doi:10.1371/journal.pone.0227428)
Supplement: S2 Table — (DOCX) [file pone.0227428.s005.docx]

| **TNL** | **Chromosome** | **Start position (bp)** | **End position (bp)** | **Annotation** |
| --- | --- | --- | --- | --- |
| **OB2-A** | 1 | 10516531 | 10526009 | Own annotation |
| **OB2-B** | 1 | 30850370 | 30854336 | Own annotation |
| **OB2-C** | 1 | 52950432 | 52954493 | Own annotation |
| **OB2-D** | 1 | 52961216 | 52965187 | Own annotation |
| **OB2-E** | 1 | 52979786 | 52984010 | Own annotation |
| **OB2-F** | 1 | 52997378 | 53001442 | Own annotation |
| **OB2-G** | 1 | 53009972 | 53013731 | Own annotation |
| **OB2-H** | 1 | 53022227 | 53026193 | Own annotation |
| **OB2-I** | 1 | 62581725 | 62585691 | Own annotation |
| **OB2-J** | 1 | 63587553 | 63583729 | Own annotation |
| **OB2-K** | 1 | 63598777 | 63595238 | Own annotation |
| **OB2-L** | 1 | 63626556 | 63622337 | Own annotation |
| **OB2-M** | 1 | 63639390 | 63636629 | Own annotation |
| **OB2-N** | 1 | 63673846 | 63669917 | Own annotation |
| **OB2-O** | 1 | 63686235 | 63682538 | Own annotation |
| **OB2-P** | 1 | 63706461 | 63702586 | RC1G0581100 ^9^ |
| **OB2-Q** | 1 | 63715560 | 63711877 | RC1G0581200 ^9^ |
| **OB2-R** | 1 | 63739819 | 63735901 | RC1G0581400 ^9^ |
| **OB2-S** | 1 | 63754856 | 63751001 | RC1G0581700 ^9^ |
| **OB2-T** | 2 | 10516531 | 10526009 | RC2G0134700 ^9^ |
| **OB2-U** | 2 | 30850370 | 30854336 | Own annotation |
| **OB1-A** | 1 | 32402135 | 32398169 | RcHm_v2.0_Chr1g0340831 ^8^ |
| **OB1-B** | 1 | 56386199 | 56390172 | RcHm_v2.0_Chr1g0365481 ^8^ |
| **OB1-C** | 1 | 56404771 | 56408995 | RcHm_v2.0_Chr1g0365531 ^8^ |
| **OB1-D** | 1 | 56435109 | 56438707 | RcHm_v2.0_Chr1g0365541 ^8^ |
| **OB1-E** | 1 | 67098739 | 67095069 | RcHm_v2.0_Chr1g0382041 ^8^ |
| **OB1-F** | 1 | 67242895 | 67238937 | RcHm_v2.0_Chr1g0382221 ^8^ |
| **OB1-G** | 5 | 69448894 | 69444877 | RcHm_v2.0_Chr5g0063811 ^8^ |
| **F.ve-1** | 7 | 16959296 | 16963184 | FvH4_7g21060.1 ^31^, FvH4_7g21060.t1 ^45^ |
| **F.ve-2** | 7 | 16964618 | 16968713 | FvH4_7g21070.1 ^31^, FvH4_7g21070.t1 ^45^ |
| **F.ve-3** | 7 | 16970642 | 16976356 | FvH4_7g21140.t5 ^45^ |
| **F.ve-4** | 7 | 16978644 | 16982408 | FvH4_7g21140.1 ^31^, FvH4_7g21140.t4 ^45^ |
| **F.ve-5** | 7 | 16988569 | 16992617 | FvH4_7g21150.1 ^31^, FvH4_7g21150.t1^45^ |
| **F.ve-6** | 7 | 17026016 | 17030059 | FvH4_7g21172.t1 ^45^ |
| **F.ve-7** | 7 | 17035014 | 17038835 | FvH4_7g21180.1 ^31^, FvH4_7g21180.t1 ^45^ |
| **F.ve-8** | 7 | 17072938 | 17075861 | FvH4_7g21230.1 ^31^, FvH4_7g21230.t2 ^45^ |
| **F.ve-9** | 7 | 23320648 | 23317230 | FvH4_7g32440.1 ^31^, FvH4_7g32440.t1^45^ |
| **F.ve-10** | 7 | 23330305 | 23326504 | FvH4_7g32460.1 ^31^, FvH4_7g32460.t1 ^45^ |
| **F.ve-11** | 7 | 23343325 | 23339511 | FvH4_7g32464.t1 ^45^ |
| **F.ve-12** | 7 | 23349582 | 23345764 | FvH4_7g32470.1 ^31^, FvH4_7g32470.t1 ^45^ |
| **F.ve-13** | 7 | 23358709 | 23354962 | FvH4_7g32480.1 ^31^, FvH4_7g32480.t1 ^45^ |
| **F.ve-14** | 7 | 23480992 | 23477145 | FvH4_7g32740.1 ^31^, FvH4_7g32740.t1 ^45^ |
| **F.ve-15** | 7 | 23489394 | 23485577 | FvH4_7g32760.1 ^31^, FvH4_7g32760.t1 ^45^ |
| **F.ve-16** | 7 | 23646795 | 23650457 | FvH4_7g33140.1 ^31^, FvH4_7g33140.t1 ^45^ |
| **F.ve-17** | 7 | 23674232 | 23678087 | FvH4_7g33190.1 ^31^ |
| **F.ve-18** | 2 | 586291 | 588494 | FvH4_2g00550.1 ^31^, FvH4_2g00550.t1^45^ |
| **F.ve-19** | 1 | 6317949 | 6322143 | FvH4_1g11580.1 ^31^, FvH4_1g11580.t1^45^ |
| **P.pe-1** | 1 | 13365644 | 13368512 | Prupe.1G165300.2 ^33,34^ |
| **P.pe-2** | 1 | 45860728 | 45874245 | Own annotation |
| **P.pe-3** | 1 | 46033718 | 46028743 | Own annotation |
| **P.pe-4** | 8 | 2064834 | 2057300 | Prupe.8G022800.1 ^33,34^ |
| **P.pe-5** | 8 | 2076406 | 2072506 | Prupe.8G023100.1 ^33,34^ |
| **P.pe-6** | 8 | 2089564 | 2086134 | Prupe.8G023200.2 ^33,34^ |
| **P.pe-7** | 8 | 2117452 | 2113704 | Prupe.8G023500.1 ^33,34^ |
| **P.pe-8** | 8 | 2131622 | 2127457 | Own annotation |
| **P.pe-9** | 8 | 2145872 | 2141889 | Prupe.8G023800.2 ^33,34^ |
| **P.pe-10** | 8 | 2175126 | 2171843 | Prupe.8G023900.1 ^33,34^ |
| **P.pe-11** | 8 | 2404806 | 2408728 | Prupe.8G026500.2 ^33,34^ |
| **P.pe-12** | 8 | 2425459 | 2429414 | Prupe.8G026700.1 ^33,34^ |
| **P.pe-13** | 8 | 2443004 | 2446952 | Prupe.8G026800.4 ^33,34^ |
| **P.pe-14** | 8 | 2457114 | 2460940 | Own annotation |
| **P.pe-15** | 8 | 2472824 | 2476778 | Prupe.8G027100.1 ^33,34^ |
| **P.pe-16** | 8 | 2503953 | 2507632 | Prupe.8G027300.2 ^33,34^ |
| **P.pe-17** | 8 | 6634106 | 6638145 | Prupe.8G056700.3 ^33,34^ |
| **M.do-1** | 0 | 26772742 | 26775888 | Own annotation |
| **M.do-2** | 0 | 26812267 | 26815686 | MD00G1125900 ^32^ |
| **M.do-3** | 0 | 26821040 | 26823969 | Own annotation |
| **M.do-4** | 5 | 3838813 | 3842214 | MD05G1022800 ^32^ |
| **M.do-5** | 5 | 1670520 | 1672590 | Own annotation |
| **M.do-6** | 10 | 2562756 | 2569113 | MD10G1020900 ^32^ |
| **M.do-7** | 10 | 4452127 | 4448258 | Own annotation |
| **M.do-8** | 10 | 4466549 | 4459307 | Own annotation |
| **M.do-9** | 10 | 4503047 | 4499285 | MD10G1035000 ^32^ |
| **M.do-10** | 10 | 4559741 | 4555923 | Own annotation |
| **R.oc-1** | 1 | 5290000 | 5287738 | Own annotation |
| **R.oc-2** | 2 | 37506549 | 37502279 | Own annotation |
| **R.oc-3** | 3 | 6669206 | 6665637 | Own annotation |
| **P.mi-1** | Contig 1260 | 86905 | 90680 | Own annotation |
| **P.mi-2** | Contig 1260 | 113600 | 117391 | Own annotation |
| **P.mi-3** | Contig 563 | 176211 | 180097 | Own annotation |
| **P.mi-4** | Contig 563 | 180989 | 190778 | Own annotation |
| **P.mi-5** | Contig 113 | 122574 | 117661 | Own annotation |
| **P.mi-6** | Contig 113 | 185679 | 174531 | Own annotation |
| **P.mi-7** | Contig 113 | 197158 | 191795 | Own annotation |
| **P.mi-8** | Contig 34 | 39375 | 44565 | Own annotation |
| **P.mi-9** | Contig 34 | 99287 | 102823 | Own annotation |
| **P.mi-10** | Contig 34 | 104624 | 108196 | Own annotation |
| **P.mi-11** | Contig 162 | 143476 | 133468 | Own annotation |
