# Supplementary material for: Analysis of the Rdr1 gene family in different Rosaceae genomes reveals an origin of an R-gene cluster after the split of Rubeae within the Rosoideae subfamily
Source: PLoS One. 2020 Jan 23;15(1):e0227428. doi: 10.1371/journal.pone.0227428 (PMC6977733; doi:10.1371/journal.pone.0227428)
Supplement: Attachment — Submitted filename: Response to Reviewers Pone D 19 26378.docx [file pone.0227428.s009.docx]

**Response to Reviewers**

PONE-D-19-26378
Analysis of the Rdr1 gene family in different Rosaceae genomes reveals an origin of an R-gene cluster after the split of Rubeae within the Rosoideae subfamily
PLOS ONE

Journal Requirements:

1. When submitting your revision, we need you to address these additional requirements.

Please ensure that your manuscript meets PLOS ONE's style requirements, including those for file naming. The PLOS ONE style templates can be found at

http://www.journals.plos.org/plosone/s/file?id=wjVg/PLOSOne_formatting_sample_main_body.pdf and <http://www.journals.plos.org/plosone/s/file?id=ba62/PLOSOne_formatting_sample_title_authors_affiliations.pdf>

**We checked the formatting.**

2.  Thank you for stating the following in the Acknowledgments Section of your manuscript:

"We thank the Leibniz Universität Hannover and the INRA (Université d’Angers, SFR
419 4207 QuaSaV) for supporting this study, as well as the ‘Région Pays de la Loire’ for
420 the resequencing of eight wild species (Genorose project in the framework of RFI
421 ‘Objectif Végétal’).".

We note that you have provided funding information that is not currently declared in your Funding Statement. However, funding information should not appear in the Acknowledgments section or other areas of your manuscript. We will only publish funding information present in the Funding Statement section of the online submission form.

* **Please remove any funding-related text from the manuscript and let us know how you would like to update your Funding Statement. Currently, your Funding Statement reads as follows:**

 "The authors received no specific funding for this work.".

**We removed the funding text from the manuscript and changed the info in the funding	statement.**

Additional Editor Comments (if provided):

Reviewers agree the manuscript is technically sound but dispute its suitability for PLOS ONE. Editor has agreed it merits the publication criteria pending minor revisions, mainly those indicated by reviewer #1, for example clarifying methodology for PCR markers, grammatical errors, etc.


[Note: HTML markup is below. Please do not edit.]

Reviewers' comments:

Reviewer's Responses to Questions

**Comments to the Author**

1. Is the manuscript technically sound, and do the data support the conclusions?

The manuscript must describe a technically sound piece of scientific research with data that supports the conclusions. Experiments must have been conducted rigorously, with appropriate controls, replication, and sample sizes. The conclusions must be drawn appropriately based on the data presented.

Reviewer #1: Yes

Reviewer #2: Partly

2. Has the statistical analysis been performed appropriately and rigorously?

Reviewer #1: Yes

Reviewer #2: Yes

3. Have the authors made all data underlying the findings in their manuscript fully available?

The [PLOS Data policy](http://www.plosone.org/static/policies.action#sharing) requires authors to make all data underlying the findings described in their manuscript fully available without restriction, with rare exception (please refer to the Data Availability Statement in the manuscript PDF file). The data should be provided as part of the manuscript or its supporting information, or deposited to a public repository. For example, in addition to summary statistics, the data points behind means, medians and variance measures should be available. If there are restrictions on publicly sharing data—e.g. participant privacy or use of data from a third party—those must be specified.

Reviewer #1: Yes

Reviewer #2: No

4. Is the manuscript presented in an intelligible fashion and written in standard English?

PLOS ONE does not copyedit accepted manuscripts, so the language in submitted articles must be clear, correct, and unambiguous. Any typographical or grammatical errors should be corrected at revision, so please note any specific errors here.

Reviewer #1: Yes

Reviewer #2: Yes

5. Review Comments to the Author

Please use the space provided to explain your answers to the questions above. You may also include additional comments for the author, including concerns about dual publication, research ethics, or publication ethics. (Please upload your review as an attachment if it exceeds 20,000 characters)

Reviewer #1: This study reveals some of the evolutionary history of the extremely economically valuable Rdr1 gene family to the horticultural industry, which has been shown to confer Black Spot resistance. Their phylogenetic analysis revealed that there are two major clusters of these genes within Rosa Old Bush, and Fragaria vesca. Genes from this family tend to cluster in regions of the chromosome that are more prone to evolve.

Their findings are of value in elucidating the evolutionary history of the Rdr1 genes in Rosaceae. Understanding their location will also assist in future breeding efforts of crops within the family, which could potentially lead to improved disease resistance. Their findings reflect those of this gene family in other genera that have been previously published.

The research conducted and the conclusions made from the results are supportive and appropriate. The authors discuss the differences between hapOB1 and hapOB2 with regards to the discrepancy in the number of Rdr1 genes. There are many Rdr1 genes missing from the genome of hapOB1 that were also not successfully PCR amplified. The authors suggest that the Rdr1 genes in hapOB1 are not likely to be an artefact and were not discovered because of poor sequencing. This is not entirely convincing though, especially since the use of PacBio technology was utilized for the generation of the hapOB1 sequence data and PCR amplification was not successful for the loci. Is it possible that a deletion event(s) occurred during meiosis that was necessary for the development of the haploid line, especially since these genes are near the end of the chromosome? The data suggest that this portion of the chromosome has somehow been lost. Sequencing errors from PacBio are possible, but to also fail at being able to amplify the genes suggest that a biological event may have occurred at that point in the genome.

**The two independent haploid cell cultures were produced by two competing research groups. Attempts to contact the second group for protocols and materials were, unfortunately, not answered but we did not want to make statements about this in the manuscript.**

**However, we changed the results and discussion part in that we now discuss assembly errors and recombination events as equally likely possibilities for the paucity of Rdr1 copies in the hapOB1 genome.**

There are no methods describing how PCR markers were used to amplify the genes in the Old Bush genomes, nor was there sequence information of the primers themselves.

**The PCR markers and methods are described in the paragraph “Analysis of the Rdr1LRR” stating that the analysis was done as described in reference [3]: Terefe D, Debener T. An SSR from the leucine-rich repeat region of the rose *Rdr1* gene family. In this publication, the primer sequences are also provided.**

Also, the authors should discuss (at least in the introduction) how and where hapOB1 and hapOB2 were generated and why DNA is not available from hapOB1.

**A short statement, on how the haploid lines have been generated, was added to the introduction.**

Also, if hapOB1 DNA is not available, how did the authors perform PCR to screen for the Rdr1 genes in the first place? Are these haploid lines available? If so, from where?

**We did not perform PCR with the HapOB1 DNA or tissue. This misunderstanding was probably caused by our complex sentence in the manuscript (now in lines 150 -155 of the manuscript with tracked changes). Therefore, we changed this sentence.**

More work could be done to clarify the introduction to the paper. It provides many details about TNL genes across the plant kingdom (which probably could be scaled back some) but doesn’t give the paper much direction as far as what specific questions the authors were trying to answer during their research or how their findings will fill in an important knowledge gap in Rosaceae. As stated before, they could discuss the origins of hapOB1/2 in the intro to make their work more understandable by non-specialists.

We added short statements to the introduction trying to explain that we wanted to extend previous studies on rosaceous NBS-LRR genes by a more detailed analysis of a single gene family including positional information of clusters and individual genes.

There are a couple of minor grammatical errors to note:
Line 60 change “between” to “among”
Line 84 change “an” to “a”

**We changed that.**

Reviewer #2: The authors describe an effort to clarify the evolutionary history of an important gene family in Rosaceae. Leveraging new genomic resources they collected sequences for a genomic region that has clusters of TNL genes at conserved locations in Rosaceae, specifically focusing on Rose. The evolutionary history of this gene family in specific lineages of Rosaceae seems to be the main message, however the broader context and relevance for either the field of evolutionary genomics or Rosaceous horticulture is not clear.

The introduction is a carefully curated collection of facts about Rose phylogeny (it's complicated) and TNLs in plants, even going as far as delving into gene intron/exon structure and patterns and patterns of genome structure in other plant lineages. Then this section abruptly ends with a declaration that the authors were interested in looking more closely at this gene family in Rose. It's not at all clear what this work helps us understand - I can think of some reasons, especially with regard to understanding the role of these important R-genes in Rose, and how this information could be very valuable to breeders, or how understanding the dynamics of the changes seen in this gene family can help us learn about how genomes evolve. This information, the potential impact of the work, or clear hypotheses, are lacking from the intro, which leaves the reader asking "so what?" from the very beginning.

**As mentioned in an answer to reviewer 1 we tried to make our research questions clearer**

**for the reader.**

Technically I think the work is sound, though admittedly my background is not strong in methods of building phylogenies. I recommend that someone with a strong background in phylogenetics/phylogenomics weigh in on the appropriateness of methods. I would think that more bootstrap reps were needed for these challenging phylogenies, and there needs to be a better way of indicating bootstrap support at critical tree positions besides triangles that vary slightly in size.

**In our analyses we used 500 bootstrap replicates. We think, that most probably additional 500 bootstraps will not result in major differences of the values.**

**The triangles of different size were used, because the font size of the real bootstrap values at the nodes would have been too small to read. We also mentioned in each figure caption below which bootstrap the branches were collapsed and the smallest bootstrap value in the dendrogram (this is also shown at this specific node):**

**Fig2: Branches reproduced in less than 75 % of bootstrap replicates are collapsed.**

**smallest Bootstrap value represents 82 %**

**Fig 3: . Branches reproduced in less than 60 % of bootstrap replicates are collapsed.**

**smallest Bootstrap value represents 70 %**

**Fig 4: Branches reproduced in less than 75 % of bootstrap replicates are collapsed.**

**smallest Bootstrap value represents 76 %,**

The context that would make this work appealing to a broad audience (i.e. that of PONE) is lacking. The conclusions drawn are appropriate (especially if another reviewer can vet the methods), but why it's important for anyone except a narrow audience is not clear, and even then, these potential narrow audiences are not addressed. The authors could write to perhaps ornamental horticulturalists, or Rose breeders, or Rose taxonomists - as it is now it's not written for the audience of PLOSONE.

The written english is technically good, the figures are OK, yet the organization and narrative quality need work. The figures are referenced out of order, which causes confusion. Table S3 is first, and it's not a Table, it' a data file as are Tables S4 and S5 - these are alignments, they should be provided as data files. Table S1 has an empty tab with labeled Tabelle3.

**We changed Table S3, 4, 5 to Datafile S3, S4 and S5. We also removed the empty tab from	Table S1.**

Overall I think the work is sound, but the presentation needs a lot of work. Critically, the authors would need to develop a narrative with broad appeal for PONE. If they decide to target a more narrow audience, they still need to work on developing a clear message and context. The authors themselves say this is a first small step, and mainly confirm prior results and persistant questions in the discussion.

6. PLOS authors have the option to publish the peer review history of their article ([what does this mean?](https://journals.plos.org/plosone/s/editorial-and-peer-review-process#loc-peer-review-history)). If published, this will include your full peer review and any attached files.


If you choose “no”, your identity will remain anonymous but your review may still be made public.


**Do you want your identity to be public for this peer review?** For information about this choice, including consent withdrawal, please see our [Privacy Policy](https://www.plos.org/privacy-policy).

Reviewer #1: No

Reviewer #2: No


[NOTE: If reviewer comments were submitted as an attachment file, they will be attached to this email and accessible via the submission site. Please log into your account, locate the manuscript record, and check for the action link "View Attachments". If this link does not appear, there are no attachment files to be viewed.]


While revising your submission, please upload your figure files to the Preflight Analysis and Conversion Engine (PACE) digital diagnostic tool, <https://pacev2.apexcovantage.com/>. PACE helps ensure that figures meet PLOS requirements. To use PACE, you must first register as a user. Registration is free. Then, login and navigate to the UPLOAD tab, where you will find detailed instructions on how to use the tool. If you encounter any issues or have any questions when using PACE, please email us at [figures@plos.org](mailto:figures@plos.org). Please note that Supporting Information files do not need this step.

*In compliance with data protection regulations, you may request that we remove your personal registration details at any time. [(Remove my information/details)](https://www.editorialmanager.com/pone/login.asp?a=r). Please contact the publication office if you have any questions.*
